# Preoperative Nutrition Support May Reduce the Prevalence of Postoperative Pancreatic Fistula after Open Pancreaticoduodenectomy in Patients with High Nutritional Risk Determined by NRS2002

**DOI:** 10.1155/2021/6691966

**Published:** 2021-05-20

**Authors:** Jing-Yong Xu, Xiao-Dong Tian, Jing-Hai Song, Jian Chen, Yin-Mo Yang, Jun-Min Wei

**Affiliations:** ^1^Department of General Surgery, Beijing Hospital, National Center of Gerontology, Institute of Geriatric Medicine, Chinese Academy of Medical Sciences, Beijing 100730, China; ^2^Department of General Surgery, Peking University First Hospital, Beijing 100034, China

## Abstract

**Background:**

Clinically relevant postoperative pancreatic fistula (CR-POPF) is a severe complication which may be caused by a perioperative nutrition problem. We aimed to study whether patients with high nutritional risk (NRS2002 score ≥ 5) might benefit from preoperative nutrition support regarding the risk of CR-POPF after open pancreaticoduodenectomy.

**Methods:**

Consecutive patients undergoing open pancreaticoduodenectomy with complete record of NRS2002 at two Chinese institutions between 2013 and 2018 were analysed. CR-POPF was diagnosed following the 2016 ISGPS criteria. Nutrition support included oral nutrition supplement and enteral and parenteral nutrition. Clinical and economic outcomes were analysed.

**Results:**

522 cases were included. 135 cases (25.9%) were at high nutritional risk (NRS2002 score ≥ 5), among which 41 cases (30.4%) received preoperative nutrition support. The CR-POPF rate was significantly lower in the preoperative nutrition support group compared with the no nutrition support group (12.2% versus 28.7%, *P* = 0.038). Multivariate analysis showed that preoperative nutrition support was a protective factor for CR-POPF in patients at high risk [OR 0.339, 95% CI (0.115-0.965), *P* = 0.039]. Higher albumin and a larger diameter of the main pancreatic duct were found to be other protectors for CR-POPF.

**Conclusions:**

Patients with high nutritional risk (NRS2002 score ≥ 5) may profit from preoperative nutritional support manifested in the reduction of CR-POPF.

## 1. Introduction

The clinically relevant postoperative pancreatic fistula (CR-POPF) is one of the major causes of morbidity after pancreaticoduodenectomy (PD) [1–3], and many risk factors were reported including preoperative factors such as jaundice, pathologic factors such as the pancreatic duct width, and nutritional factors such as body mass index (BMI) and malnutrition [4, 5]. However, an international survey showed that 44% of surgeons did not implement a preoperative nutritional consultation for their patients undergoing PD, and no specific preoperative nutritional thresholds were used [6].

Actually, a nutrition care plan contains three steps: nutritional screening, nutrition assessment, and nutrition intervention [7]. Nutritional screening is the first step [8]. As it was recommended in guidelines from the European Society of Nutrition and Metabolism (ESPEN) [9] and the American Society of Parenteral and Enteral Nutrition (ASPEN) [10], the screening tool Nutritional Risk Screening 2002 (NRS2002) is the first choice of screening because it is evidence-based and validated by both retrospective and prospective studies [11, 12]. Some studies in gastrointestinal surgery showed that preoperative nutrition support might improve the postoperative outcomes in the patients with nutritional risk (NRS2002 score ≥ 3) [13], but no data were reported in the field of pancreatic surgery [14]. What is more, the prognostic value of different nutritional assessment scores in pancreatic surgery is still controversial [15].

In China, NRS2002 is the first choice for nutrition screening. The aim of this retrospective study is to analyse whether Chinese patients with nutritional risk screened by NRS2002 may benefit from preoperative nutrition support.

## 2. Materials and Methods

### 2.1. Patients and Baseline Characteristics

Data of consecutive patients undergoing open PD at two university hospitals in China between May 2013 and May 2018 were analysed retrospectively. Approval from the local ethics committee includes the usage and publication of these retrospectively analysed data. Due to the blinded data and retrospective design, written informed consent was not considered necessary by the ethics committee (Approval letter No. 2018BJYYEC-196-02).

Baseline variables such as age, sex, body mass index (BMI), ASA classification, jaundice, diabetes, coronary artery disease, hypertension, smoking, and drinking were recorded. Preoperative blood tests contained white blood cell count (WBC), haemoglobin (Hb), albumin (Alb), AST, ALT, total bilirubin (TBil), direct bilirubin (DBil), serum amylase (AMY), APTT, PT, and CA19-9.

### 2.2. Nutritional Management

NRS2002 was used as the screening tool within 24 hours after admission. NRS2002 contains three parts: (1) score for nutritional status (0 to 3 scores, containing weight loss, declined intake, and low BMI), (2) score for severity of disease (0 to 3 scores, representing different degree of stress metabolism and increased nutritional requirements), and (3) score for age (1 score for patients 70 years or older). The total score ranges from 0 to 7. If the NRS2002 score is more than 3, it means “at nutritional risk” [16]. If the NRS2002 score is more than 5, it means “at high nutritional risk,” while 3 and 4 scores are “at low nutritional risk” [17].

Score for severity of disease in our study was 2 scores in all patients due to the major open abdominal operation. So in the high risk (NRS2002 ≥ 5) group, there are three subgroups of score constitution: (1) subgroup 1 (first type of 5 scores): nutrition impairment = 2, disease severity = 2, and age = 1; (2) subgroup 2 (second type of 5 scores): nutrition impairment = 3, disease severity = 2, and age = 0; (3) subgroup 3 (6 scores): nutrition impairment = 3, disease severity = 2, and age = 1. In order to reduce the selection bias and prevent the imbalance of basal data in the support and no support group, we did comparisons among different subgroups.

Nutrition support therapy in this study contained oral nutrition supplement (ONS), enteral nutrition (EN), and parenteral nutrition (PN) implemented for more than seven days which were defined as “nutrition support” and studied in this paper [18, 19]. PN contained total PN (TPN) and supplementary PN (SPN). The target of the protein was 1-1.2 g/kg/d, and the energy reached at least 70% of the daily requirement (20-25 kcal/kg/d).

**Figure 1:**
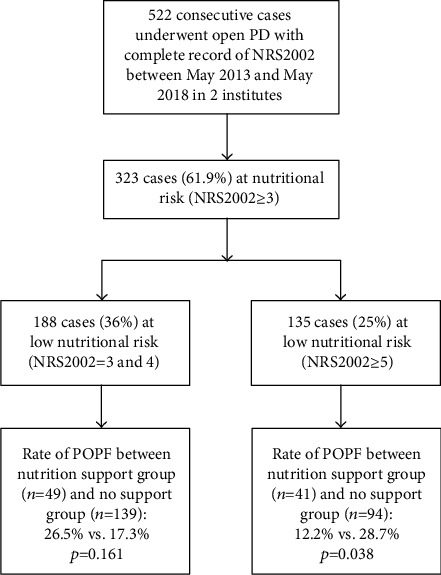
Flow chart of the study.

Since this is a retrospective study, and due to the lack of attention of preoperative nutrition support, the application of preoperative nutrition to the patients was not entirely based on the result of NRS2002 but the surgeon's discretion in accordance with the nutrition status, which provided us a chance to do a “real-world” judgement whether preoperative nutritional support was effective. So the patients at nutritional risk could be divided into two groups: preoperative nutrition support group and no support group.  Figure 1 shows the flowchart of the study design. Postoperative nutrition support was administrated according to the guidelines.

### 2.3. Operative Data and CR-POPF

The operative procedures and perioperative management are unified in these two hospitals because of long-term collaboration [20]. Operative details include the method of pancreaticojejunostomy (duct-to-mucosa or invagination anastomosis), volumes of blood loss, and intraoperative fluid infusion. Some morphologic characters were also recorded such as diameter of the main pancreatic duct (diameter > 3 mm was defined as large duct), texture of the pancreas (hard or soft), and pathologic diagnosis. CR-POPF was defined and graded according to the 2016 ISGPS classification [21]. Only clinically relevant CR-POPF (grades B and C) were included, and both normal and biochemical leak cohorts were defined as the non-CR-POPF group. Whenever a CR-POPF occurred, the treatment depended on the institutional practices and patients' condition.

### 2.4. Clinical and Economic Outcome Measures

We defined that all postoperative outcomes recorded happened until discharge. Besides CR-POPF, nonfistulous complications like cardiac and cerebrovascular events, postoperative haemorrhage (PPH), biliary fistula, abdominal infection, wound infection, and delayed gastric emptying (DGE) were also analysed. We followed the ISGPS definitions and classifications of PPH and DGE [22, 23]. Postoperative length of hospital stay (LOS), 30-day readmission rate, perioperative mortality, and total hospital costs were all recorded in CR-POPF groups. Total hospital costs only contained the direct cost drawn from the bill of hospitalization expenses before the reimbursement of insurance, which mainly included fees for hospital and nursing care, operation and other professional services, use of medical equipment, and prescription drugs.

### 2.5. Statistical Analysis

The data were collected and checked by two staff to ensure accuracy at the two institutions. IBM SPSS Statistics (Ver. 20.0, IBM Corp., Armonk, NY, USA) was used to do the statistical analysis by professional statisticians. Categorical data were analysed using the chi-square test or Fisher exact test. Continuous data was tested by Student's unpaired *t* test. A multivariable logistic regression model was used to evaluate the relationship between risk factors and outcomes, which was expressed as an odds ratio (OR) with 95% confidence intervals. According to recent articles, we chose several most commonly used factors to do the logistic regression analysis, including age, BMI, pancreatic duct width, method of pancreaticojejunostomy, and implantation of pancreatic duct stenting [24–26]. Two nutrition-related factors were included: preoperative albumin and nutrition support. *P* values of less than 0.05 were considered statistically significant.

## 3. Results

### 3.1. Basal Data of All Patients

In total, 522 consecutive cases who underwent open pancreaticoduodenectomy were included. The male : female ratio was 1.5 : 1 (309 : 213). The mean age was 62.0 ± 11.6 years (range 16–88 years). 129 cases (24.7%) had diabetes, 180 cases (34.5%) had hypertension, and 46 cases (8.8%) had coronary heart disease. 151 patients (28.9%) were heavy drinkers, and 173 patients (33.1%) were heavy smokers.

The operative data showed that the blood loss was 372.9 ± 118.3 mL, and the average operation time was 363.8 ± 108.2 min. The median diameter of the main pancreatic duct was 3.1 ± 2.0 mm. 506 cases had complete records of the way of pancreaticojejunostomy, in which 228 (45.1%) were invagination anastomosis and 278 (54.9%) were duct-to-mucosa anastomosis, with no significant difference in the CR-POPF rate between these two groups (24.1% vs. 19.6%, *P* = 0.378). Pancreatic duct stents were preserved in 276 cases (52.9%), but there was no difference in the prevalence of CR-POPF with the nonstent cases (18.5% vs. 23.7%, *P* = 0.311).

### 3.2. Patients at High Nutritional Risk (*NRS*2002 *Score* ≥ 5)

135 cases were at high nutritional risk (NRS2002 score ≥ 5) and 41 cases (30.4%) received preoperative nutrition support, among which 36 cases were on TPN or diet with SPN, and 5 cases were on diet with ONS or diet with EN. No nutrition support-related complications were recorded. The baseline data are presented in Table 1. Basal data such as age, sex, body mass index, historic, laboratory, perioperative data, and pathologic diagnosis were comparable between the two groups. Notedly, only six people have albumin below 30 g/L.  Table 2 shows that no difference was found in the NRS2002 score constitution in the preoperative nutrition support group and no support groups in patients at high nutritional risk.

The comparison between the prevalence of common complications after PD and outcomes is shown in Table 3. The prevalence of CR-POPF was 23.7% (32/135) and the CR-POPF rate was significantly lower in the preoperative nutrition support group compared with no nutrition support group (12.2% versus 28.7%, *P* = 0.038). Except for this, no statistic differences were found in the other complications. The rates of postoperative length of stay, 30-day readmission rate, mortality, and total hospital costs were not significantly different between the two groups.

Multivariate logistic analysis showed preoperative nutrition support as a protective factor for CR-POPF in patients at high nutritional risk [OR 0.339, 95% CI (0.115-0.965), *P* = 0.039]. Meanwhile, high albumin was proven to be related to a lower rate of CR-POPF [OR 0.910, 95% CI (0.834-0.994), *P* = 0.037], and a larger diameter of the main pancreatic duct might be another protector for CR-POPF [OR 0.449, 95% CI (0.253-0.797), *P* = 0.006] (Table 4).

### 3.3. Patients at Risk (*NRS*2002 ≥ 3) and at Low Risk (*NRS*2002 = 3 and 4)

In total, 323 cases (61.9%) were at nutritional risk, among which only 86 cases (26.6%) received preoperative nutrition support. The rate of CR-POPF of patients at nutritional risk (NRS2002 ≥ 3) was 21.4% (69/323). 188 cases were at low nutritional risk (NRS2002 = 3 and 4) with 45 cases receiving preoperative nutrition support. The rate of CR-POPF was 19.7% (37/188). The prevalence of CR-POPF was comparable between nutrition support and no support cohorts in both at-risk patients and at low-risk patients (Table 5).

## 4. Discussion

It was also recommended by ISGPS in 2018 that all patients who undergo pancreatic operation should receive nutritional screening and assessment to determine the nutrition status and the indication of perioperative nutritional care [14]. Nutritional screening is the first step of nutrition care. Many tools could be chosen including NRS2002, MNA-SF, and MUST, but the result of different tools differs [27]. The result of NRS2002 is “nutritional risk” which was defined as the risk of developing adverse outcome due to nutrition problems but not risk of developing malnutrition. NRS2002 is the only tool that originated from evidence-based medicine and was validated by both retrospective and prospective studies [28]. NRS2002 contains the evaluation of weight loss, BMI, reduction of food intake, severity of disease, and age, each of which was proven to be risk factors of postoperative complications of pancreatectomy [29, 30]. However, as a comprehensive tool, it has been doubted in a recent paper by Probst et al. from Heidelberg that none of the malnutrition defined by the available nutritional screening and assessment tools related to postpancreatectomy complications and he suggested abandoning these scores [15]. In our opinion, the authors neglected a core point that if the patients at nutritional risk received standard nutrition support, the outcome might be improved, which might lead to no significant difference between at-risk and not-at-risk cohorts, and this is exactly the critical role nutrition screening and assessment tools played in nutrition care—to indicate nutrition support. Therefore, whether to abandon them is questionable. So in our study, we compared the prevalence of complication and outcome between the nutrition support group and the no support group in different risk score levels and proved the protective role of preoperative nutrition support in reducing the rate of CR-POPF in the patients at high nutritional risk determined by NRS2002. The same conclusion of reducing infectious complications was confirmed in major abdominal surgery including pancreatectomy and cited by two American guidelines [10, 13, 17].

Current published articles mainly focus on postoperative nutritional support, but there are no objective data or guidelines specifically addressing the need for preoperative nutritional support for patients undergoing pancreatic surgery [14]. In 2017, the ESPEN guideline on surgery recommends 7-14 days of nutritional support prior to major GI surgery in malnourished patients who fulfil one of the following criteria: weight loss > 10–15% within 6 months; BMI < 18.5 kg/m^2^; Subjective Global Assessment (SGA) Grade C or NRS2002 > 5; and preoperative serum albumin < 30 g/L (with no evidence of hepatic or renal dysfunction) [9]. In 2018, the ISGPS guideline cited the ESPEN guidelines and recommended preoperative nutritional support in severely malnourished patients [14]. However, these parameters were validated only in gastrointestinal surgery other than pancreatic surgery. So in our study, we proved in the field of pancreatectomy that high nutritional risk (NRS2002 score ≥ 5) should be the validated indication of preoperative nutrition support in order to reduce CR-POPF.

Albumin was proven to be an independent risk factor of CR-POPF in our study. However, only six cases have albumin below 30 g/L in the high risk group before operation. Due to the small number of cases, further statistical analysis is not possible to determine the cut-off value in our study. But on the other hand, albumin may not be a tool to judge nutritional problems, but it is a parameter of inflammation, which has been recommended by the 2016 American College of Gastroenterology clinical guideline of nutrition therapy in the adult hospitalized patient that “traditional” nutrition indicators such as albumin should be avoided for nutritional assessment [10].

There are several limitations to the present study. Firstly, this is a retrospective study; all we studied were drawn from medical records. Secondly, the sample size is relatively small to further analyse some details and stratification such as age, albumin, and nutrition support route. Thirdly, we might see that except for CR-POPF, postoperative haemorrhage, abdominal infection, postoperative LOS, and total hospital costs did not differ between the preoperative nutrition support and no support groups. We thought it was mainly due to the small sample size and low prevalence of these problems. And we could see a trend in Table 3 that in most other items, the results in the no-support group were worse than those in the support group though no statistical significance was found. Last, the allocation was not based on NRS2002; it was based on the judgment of the surgeon which is the current status of nutrition support in China which needs further unification. So, a prospective study with more cases and centers should be undertaken to further validate the guidance value of the tool.

In conclusion, the NRS2002 is simple way to implement a preoperative nutritional assessment before PD in order to prevent the risk of POPF. Patients with high nutritional risk may profit from preoperative nutrition support manifested in decreasing the risk of CR-POPF.

## Figures and Tables

**Table 1:** Basal data in preoperative nutrition support and no support groups in patients at high risk (NRS2002 score ≥ 5).

Variable	Preoperative nutrition support group (*n* = 41)	No support group (*n* = 94)	*P*
Age, mean (SD) (y)	60.2 (14.3)	63.8 (11.1)	0.117
Sex, male, *n* (%)	22 (53.7)	46 (48.9)	0.614
BMI, mean (SD) (kg/m^2^)	22.8 (4.1)	23.7 (3.3)	0.169
History, *n* (%)			
Diabetes	8 (19.5)	30 (31.9)	0.141
Hypertension	11 (26.8)	39 (41.5)	0.105
Coronary heart disease	4 (9.8)	10 (10.6)	0.877
Smoking	13 (31.7)	23 (24.5)	0.382
Drinking	12 (29.3)	18 (19.1)	0.193
Jaundice	24 (58.5)	44 (46.8)	0.210
Laboratory			
Albumin, mean (SD), (g/L)	38.5 (6.6)	37.9 (4.7)	0.572
White blood cell count, mean (SD) (×10^9^/L)	6.7 (3.4)	6.1 (2.7)	0.276
Haemoglobin, mean (SD) (g/L)	119.8 (18.0)	125.6 (17.0)	0.082
ALT, mean (SD) (U/L)	165.2 (163.7)	162.2 (157.1)	0.081
TBIL, mean (SD) (mg/dL)	120.6 (114.3)	87.4 (110.8)	0.116
AMY, mean (SD) (U/L)	77.1 (77.3)	66.0 (57.7)	0.550
Operation			
Operation duration, mean (SD) (min)	373.3 (92.7)	359.6 (114.6)	0.557
Intraoperative blood loss, mean (SD) (mL)	748.8 (766.1)	524.5 (560.3)	0.501
Intraoperative fluid infusion, mean (SD) (mL)	3406.1 (1416.4)	3658.6 (1727.9)	0.413
Diameter of main pancreatic duct, mean (SD) (mm)	3.3 (1.8)	3.0 (2.1)	0.577
Duct-to-mucous pancreaticojejunostomy, *n* (%)	25 (61.0)	60 (63.8)	0.752
Hard pancreatic texture, *n* (%)	21 (51.2)	38 (40.4)	0.245
Pancreatic duct stent, *n* (%)	32 (78.0)	59 (62.8)	0.081
Pathology, *n* (%)			0.530
Pancreatic duct adenocarcinoma	8 (19.5)	27 (28.7)	
Cholangiocarcinoma	12 (29.3)	26 (27.7)	
Ampullary carcinoma	9 (22.0)	14 (14.9)	
Duodenal carcinoma	3 (7.3)	7 (7.4)	
Other malignances	4 (9.8)	7 (7.4)	
Benign tumors	1 (2.4)	7 (7.4)	
Chronic pancreatitis	2 (4.8)	5 (5.3)	
Others	2 (4.8)	1 (1.1)	

**Table 2:** Comparison of NRS score constitution in preoperative nutrition support and no support groups in patients at high risk (NRS2002 score ≥ 5).

NRS score constitution	Total cases (*n* = 135)	Preoperative nutrition support group (*n* = 41)	No support group (*n* = 94)	*P*
Subgroup 1, *n* (%)	27 (20)	6 (14.6)	21 (22.3)	0.303
Subgroup 2, *n* (%)	100 (74.1)	31 (75.6)	69 (73.4)	0.788
Subgroup 3, *n* (%)	8 (5.9)	4 (9.8)	4 (4.3)	0.213

Subgroup 1: 5 scores—nutrition impairment = 2, disease severity = 2, and age = 1; Subgroup 2: 5 scores—nutrition impairment = 3, disease severity = 2, and age = 0; Subgroup 3: 6 scores—nutrition impairment = 3, disease severity = 2, and age = 1.

**Table 3:** Complication and outcome comparison between preoperative nutrition support and no support groups in patients at high risk (NRS2002 score ≥ 5).

Variable	Preoperative nutrition support group (*n* = 41)	No support group (*n* = 94)	*P*
Complications, *n* (%)			
CR-POPF	5 (12.2)	27 (28.7)	0.038
Postoperative haemorrhage	5 (12.2)	13 (13.8)	0.797
Biliary fistula	2 (4.9)	11 (11.7)	0.358
Abdominal infection	6 (14.6)	8 (8.5)	0.444
Wound infection	2 (4.9)	4 (4.3)	0.872
Delayed gastric emptying	6 (14.6)	15 (16.0)	0.845
Cardiac and cerebrovascular events	0 (0)	1 (1.1)	0.394
Outcomes			
Postoperative LOS, mean (SD) (day)	26.9 (14.6)	26.0 (20.0)	0.788
30-day readmission rate, *n* (%)	2 (4.9)	0 (0.0)	0.091
Perioperative mortality, *n* (%)	1 (2.4)	3 (3.2)	0.809
Total hospital costs, mean (SD) (USD)	20238.1 (12059.3)	21796.8 (14451.0)	0.617

**Table 4:** Univariate and multivariate analysis of CR-POPF rate in patients at high nutritional Risk (NRS2002 score ≥ 5).

Variable	Univariate		Multivariate	
	OR (95% CI)	*P*	OR (95% CI)	*P*
Age	1.039 (1.000-1.079)	0.050		
BMI	1.106 (0.990-1.235)	0.074		
Albumin	0.914 (0.839-0.996)	0.039	0.910 (0.834-0.994)	0.037
Nutritional support therapy	0.345 (0.122-0.972)	0.044	0.339 (0.115-0.965)	0.039
Diameter of main pancreatic duct	0.469 (0.265-0.830)	0.009	0.449 (0.253-0.797)	0.006
Pancreatic texture	0.501 (0.216, 1.162)	0.107		
Duct-to-mucous pancreaticojejunostomy	1.974 (0.857-4.547)	0.110		
Pancreatic duct stent	2.052 (0.990-5.073)	0.143		

**Table 5:** CR-POPF and outcome comparison between preoperative nutrition support and no support groups in all patients at risk (NRS2002 score ≥ 3) and low risk (NRS2002 score = 3 and 4).

Variable	Preoperative nutrition support group	No support group	*P*
Patients at risk (NRS2002 score ≥ 3)	*n* = 90	*n* = 233	
CR-POPF, *n* (%)	18 (20.0)	51 (21.9)	0.710
Postoperative LOS, mean (SD) (day)	26.9 (14.0)	25.8 (18.1)	0.592
30-day readmission rate, *n* (%)	2 (2.2)	2 (0.9)	0.310
Perioperative mortality, *n* (%)	1 (1.1)	7 (3.0)	0.560
Total hospital costs, mean (SD) (USD)	21886.0 (12396.0)	21677.3 (12107.3)	0.904
Patients at low risk (NRS2002 score = 3 and 4)	*n* = 49	*n* = 139	
CR-POPF, *n* (%)	13 (26.5)	24 (17.3)	0.161
Postoperative LOS, mean (SD) (day)	26.9 (13.7)	25.7 (16.8)	0.634
30-day readmission rate, *n* (%)	0 (0.0)	2 (1.4)	0.546
Perioperative mortality, *n* (%)	0 (0.0)	4 (2.9)	0.118
Total hospital costs, mean (SD) (USD)	23039.5 (12648.6)	21608.9 (10602.9)	0.485

## Data Availability

The retrospective data used to support the findings of this study are restricted by the ethics boards of both hospitals of the corresponding authors in order to protect patient privacy. Some of the data may be available from the corresponding author (Jun-Min Wei) upon request.
